# CESNET-TimeSeries24: Time Series Dataset for Network Traffic Anomaly Detection and Forecasting

**DOI:** 10.1038/s41597-025-04603-x

**Published:** 2025-02-26

**Authors:** Josef Koumar, Karel Hynek, Tomáš Čejka, Pavel Šiška

**Affiliations:** 1 CESNET, Generála Píky 430/26, 160 00 Prague 6, Czech Republic; 2 Czech Technical University in Prague, Thákurova 9, 160 00 Prague 6, Czech Republic

**Keywords:** Scientific data, Information technology

## Abstract

Anomaly detection in network traffic is crucial for maintaining the security of computer networks and identifying malicious activities. Most approaches to anomaly detection use methods based on forecasting. Extensive real-world network datasets for forecasting and anomaly detection techniques are missing, potentially causing overestimation of anomaly detection algorithm performance and fabricating the illusion of progress. This manuscript tackles this issue by introducing a comprehensive dataset derived from 40 weeks of traffic transmitted by 275,000 active IP addresses in the CESNET3 network—an ISP network serving approximately half a million customers daily. It captures the behavior of diverse network entities, reflecting the variability typical of an ISP environment. This variability provides a realistic and challenging environment for developing forecasting and anomaly detection models, enabling evaluations that are closer to real-world deployment scenarios. It provides valuable insights into the practical deployment of forecast-based anomaly detection approaches.

## Background & Summary

Network traffic monitoring plays a crucial role in network management and overall computer security^1^. Network-based intrusion detection and prevention systems can protect infrastructure against users’ sloppiness, policy violations, or intentional internal attacks. The widespread adoption of traffic encryption significantly reduces visibility, making the maintenance of network security challenging.

As a result, gaining insights into encrypted network traffic has become essential, particularly for threat detection. Recent research has focused on detecting security threats through the classification of encrypted traffic using machine learning techniques^2–6^. Supervised machine learning classification models can detect known attacks captured in the training dataset or those resembling them (e.g. malware from the same family). However, in the network traffic monitoring domain, there is a substantial challenge in acquiring up-to-date threat datasets^7^. Therefore, unsupervised anomaly detection plays a crucial role in network traffic monitoring^8^ as it can detect unknown (zero-day) attacks based on behavioral changes caused by infection^9^.

The unsupervised anomaly detection method assigns anomalous scores to network entity behavior based on patterns and characteristics learned from historical data. One of the most popular unsupervised anomaly detection algorithms is based on traffic forecasting, also referred to as network traffic prediction. An anomaly is detected by an algorithm when the difference between the forecasted value and observation exceeds a defined threshold. Traffic forecasting is useful for other networking use cases, such as data-driven network management, resource allocation, and service orchestration.

In recent years, there has been a rapid development in forecasting and anomaly detection methods, not limited to computer science. Wu *et al*.^10^ attributed this development to the rise and successful use of neural networks. The recent performance improvement of forecasting methods for network traffic monitoring remains uncertain due to the absence of long-term datasets^11^. In their survey, Ferreira *et al*.^11^ describe the lack of a reference dataset as the crucial obstacle related to performance evaluation. Additionally, real-world datasets used in the evaluation are not publicly available due to privacy concerns. Therefore, most publicly available datasets have synthetic origins.

Synthetic data does not necessarily represent real-world use. Wu *et al*.^10^ show that novel anomaly detection and forecasting approaches evaluated on synthetic datasets lead to the illusion of nonexistent progress. The preferable option is to use up-to-date real-world data, like the MAWIlab project WIDE^12^, which publishes anonymized packet captures daily. However, the WIDE project provides only brief 15-minute daily packet traces^13^, which is an insufficient time window for effective traffic modeling.

We created the CESNET-TimeSeries24 dataset^14^ to address these challenges by including long-term monitoring of selected statistical metrics for 40 weeks for each IP address on the ISP network CESNET3 (Czech Education and Science Network). The dataset encompasses network traffic from more than 275,000 active IP addresses assigned to a wide variety of devices, including office computers, NATs, servers, WiFi routers, honeypots, and video-game consoles found in dormitories. Moreover, the dataset is also rich in network anomaly types since it contains all types of anomalies identified by Chandola *et al*. and Basdekidou *et al*.^15,16^, ensuring a comprehensive evaluation of anomaly detection methods. Last but not least, the CESNET-TimeSeries24 dataset^14^ provides traffic time series on individual IP, institutional, and institutional subnet levels to cover all possible anomaly detection or forecasting scopes. Overall, the time series dataset was created from the 66 billion IP flows that contain 4 trillion packets that carry approximately 3.7 petabytes of data. The CESNET-TimeSeries24 dataset^14^ is a complex real-world dataset that will finally bring insights into the evaluation of forecasting models in real-world environments.

## Methods

**Fig. 1:**
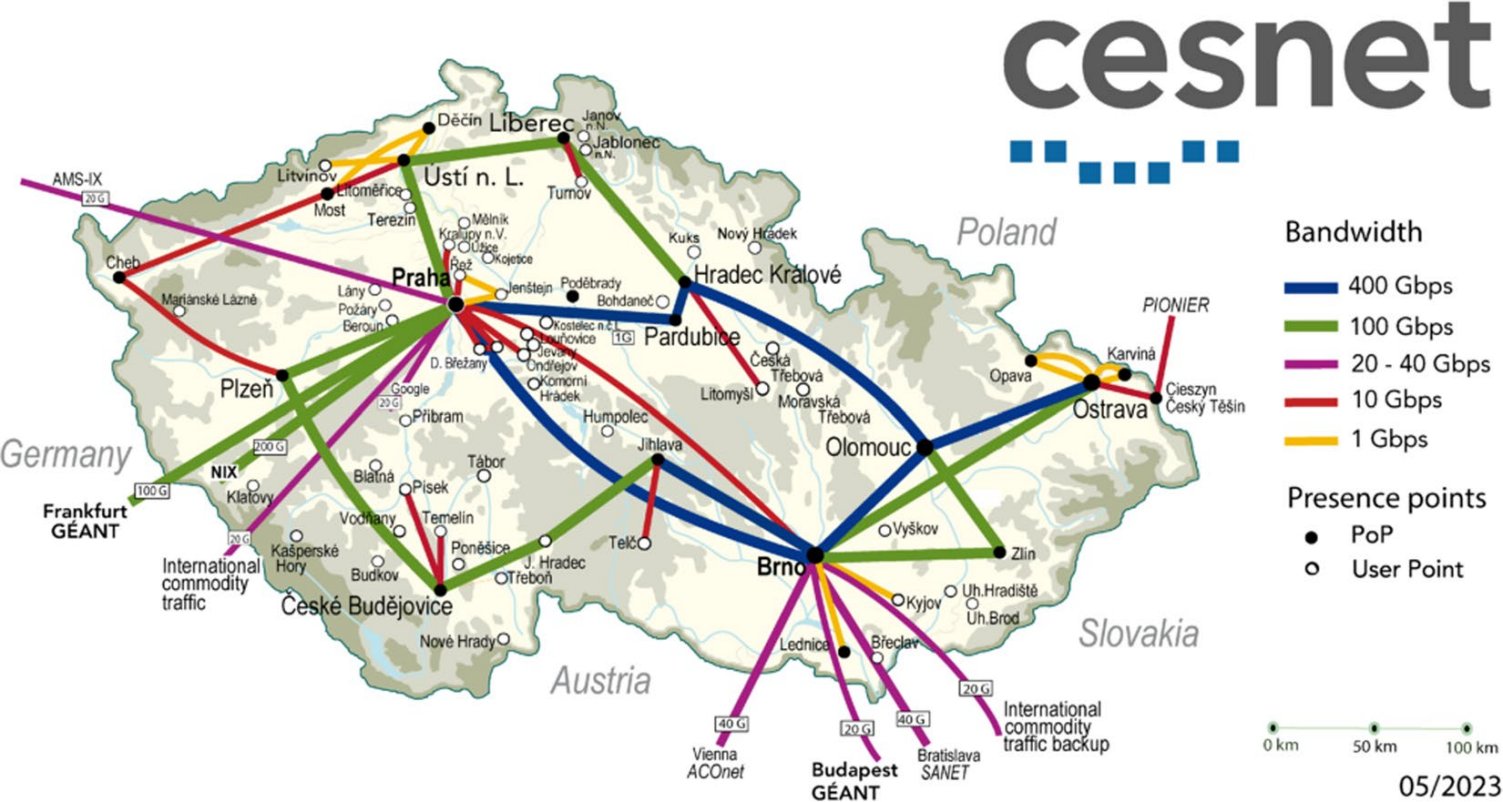
Topology of the CESNET3 network, which interconnects academic institutions in the Czech Republic.

The dataset was obtained from the network traffic of the CESNET3 network—an ISP-like network providing internet access to public and research institutions in the Czech Republic. The network spans the whole country, as shown in Fig. 1, and serves approximately half a million daily users.

### Ethics Statement

The advantage of real-world traffic data comes with a fundamental concern of privacy. We used only automatic data processing with immediate data anonymization. With this, we declare that we did not analyze or manually process non-anonymized data or perform any procedures that could allow us to track users or reveal their identities.

The Committee for Ethics in Research of the Scientific Council of the Czech Technical University in Prague approved the publication of the dataset under reference number 0000-10/24/51902/EKČVUT. The approval also includes a waiver of explicit user consent for publishing the dataset. Moreover, all users of the CESNET3 network agreed with the terms and conditions that define a monitoring process for optimization and improvement of services (including related research) and allow sharing of the data with third parties after anonymization (https://www.cesnet.cz/en/gdpr).

### Data capture

Since ISP networks transfer huge volumes of data, packet-based monitoring systems are infeasible due to the costs of processing and storage. Therefore, the CESNET3 network is monitored using a standard IP flow monitoring system located at the perimeter. All transit lines to the peering partners are equipped with flow-monitoring probes.

IP flow monitoring systems aggregate data packets into IP flow records. An IP flow record represents communication metadata associated with a single connection and is defined^17^ as “a set of IP packets passing an observation point in the network during a certain time interval, such that all packets belonging to a particular flow have a set of common properties”. These properties are commonly referred to as flow keys in related works and consist of source and destination IP addresses, transport layer ports, and protocol.

**Fig. 2:**
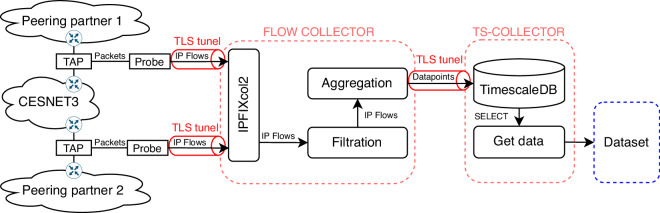
Architecture of dataset collection.

The monitoring infrastructure for creating the dataset is detailed in Fig. 2. Network TAPs are before the edge routers of the CESNET3 network and mirror the traffic to monitoring probes, which are servers equipped with a high-speed monitoring card capable of handling 200 Gb/s. The flow exporter ipfixprobe (https://github.com/CESNET/ipfixprobe) is installed on the monitoring probe. The ipfixprobe exporting process was set with an active timeout of 5 minutes and an inactive (idle) timeout of 65 seconds—it splits long connections when the connection duration is longer than the active timeout and exports a flow record even though the actual connection is not terminated yet. If ipfixprobe observes no packet within the inactive timeout period, it considers the connection terminated and exports a flow record. Using active and inactive timeouts for splitting connections is standard practice for flow-based network monitoring^17^. Additionally, the ipfixprobe collected the following features: start time, end time, number of packets, number of bytes, and Time to Live (TTL). None of the collected information contains application-level information to ensure the privacy of user communications is not compromised. The collected data are sent using IPFIX^17^ protocol and secured (TLS) tunnel to the IP flow collector server with IPFIXcol2 (https://github.com/CESNET/ipfixcol2) flow collecting software installed.

### Time series aggregation

The flow collector server contains filtration and aggregation modules as depicted in Fig. 2. The filtration module removes all transient traffic where both source and destination addresses do not belong to CESNET3, and the packets are just passing through the network. We also filter out all single TCP-SYN packet connections, i.e., scans. Since the network probes are placed before the edge routers and firewalls, TCP scans make up the majority of the collected flows. Too many scans would cause significant noise in the time series and result in possible anomaly detection false negatives. Since simple methods^18,19^ can detect the scans, we decided to exclude them from the dataset and filter them out. The filtration module passes all other flows to the aggregation module.

**Fig. 3:**
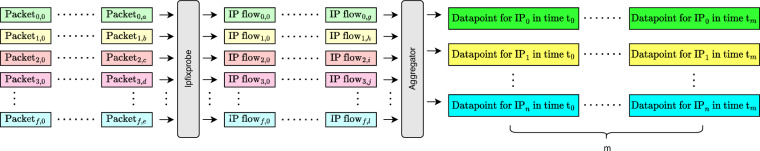
This diagram describes the aggregation process for capturing time series from network traffic.

We generate evenly spaced time series for each IP address by aggregating IP flow records into time series datapoints. The process diagram for network traffic processing and aggregation is shown in Fig. 3. The created datapoints represent the behavior of IP addresses within a defined 10-minutes time window. The vector of time-series metrics *v*_*ip*,*i*_ describes the IP address *ip* in the *i*-th time window. Thus, IP flows for vector *v*_*ip*,*i*_ are captured in time windows starting at *t*_*i*_ and ending at *t*_*i*+1_. The aggregator module sends aggregated datapoints using a secured (TLS) tunnel into the Time Series collector. The datapoints are stored in TimeScaleDB (https://www.timescale.com). Time series are built by selecting datapoints from the database.

Datapoints created by the aggregation of IP flows contain the following time-series metrics:

- ***Simple volumetric metrics:*** the number of IP flows, packets, and bytes
- ***Unique volumetric metrics:*** the number of unique destination IP addresses, destination Autonomous System Numbers (ASNs), and destination transport layer ports.
- ***Ratios metrics:*** the ratio of UDP/TCP packets, UDP/TCP transmitted data size, packets direction, and bytes direction
- ***Average metrics:*** the average flow duration, and Time To Live (TTL)

The aggregation of *Unique volumetric metrics* is memory intensive since all unique values must be stored in an array. We used a server with 41 GB of RAM, which was enough for 10-minute aggregation on the ISP network.

### Anonymization

The capturing process started on 9. October 2023. On 14. July 2024, after 40 weeks, we ended data capturing and extracted time series from the database. As part of the database framework, a script automatically adds a corresponding institution and an institution subnet for each IP address. We do not store IP addresses, institution names, or exact institution subnets. Instead, we use randomly assigned numbers as identifiers for IP addresses, institutions, and institution subnets during database creation, which performs effective anonymization. Without access to the mapping table inside the database framework, this step cannot be reverted to connect the time series with a particular IP address, institution, or institutional network.

### Data preprocesing

Preprocessing steps included: *i)* time series filtering, *ii)*creation of multiple time aggregation, *iii)* creation of time series for institutions, *iv)* creation of time series of institutional subnets, and *v)* adding information about weekends and holidays.

#### Time series filtering

The raw dataset obtained from the database has time series data for approximately 400,000 IP addresses. However, many of these network entities showed very low activity—most of the time, they were inactive, leading to largely empty records in the 10-minute aggregation. To further reduce the dataset size, we opted to remove unusable time series by filtering out those with more than 99.75% of their time intervals empty (i.e., containing 100 or fewer non-empty data points). In comparison, studies focused on forecasting with missing values often consider 50% missing data as an extreme case^20,21^. Our approach, therefore, eliminates only time series that are effectively unusable for forecasting. This filtering step reduced the dataset size to 275,124 IP addresses.

#### Multiple time aggregation

Datapoints aggregate network traffic into 10-minute intervals. The size of the aggregation interval influences anomaly detection procedures, mainly the training speed of the detection model. Recognizing 10-minute intervals can be too short for longitudinal anomaly detection methods. We provide additional one-hour and one-day aggregation intervals. These intervals were aggregated from the 10-minute interval using the sum of values. In certain cases, such as with metrics that contain a number of unique values, a simple summation would result in information loss. To address this, we provide three additional time-series metrics for each original one: sum, mean, and standard deviation. Finally, we use the mean for the rest of the time series metrics. Metrics aggregation methods are shown in Table 1.

**Table 1:** Metrics aggregation methods.

Metric	Aggregation method
Number of IP flows	sum
Number of packets	sum
Number of bytes	sum
Number of unique destination IP addresses	sum, mean, and standard deviation
Number of unique destination ASNs	sum, mean, and standard deviation
Number of unique destination transport layer ports	sum, mean, and standard deviation
Ratio of UDP/TCP packets	mean
Ratio of UDP/TCP transmitted data size	mean
Direction ratio of packets	mean
Direction ratio of bytes	mean
Average flow duration	mean
Average TTL	mean

#### Time series of institutions

We use the 283 unique institution IDs exported from the database to group the IP addresses based on institution. These can provide the overall network traffic perspective required for the identification of many security events.

#### Time series of institutional subnets

Many institutions have multiple networks, or subnets, for different locations or for different purposes in the organization. Network administrators often handle security for the overall network and per subnet. To accommodate that, we divide the IP addresses into groups based on institution subnets in the same location using information from the CESNET association. We identify 548 institution subnets inside the CESNET3 network.

#### Weekend and holidays

Network traffic is different on weekends and holidays making it crucial for forecasting and anomaly detection model training and evaluation. To support this, we included weekend dates and dates of public holidays in the Czech Republic.

## Data Records

**Fig. 4:**
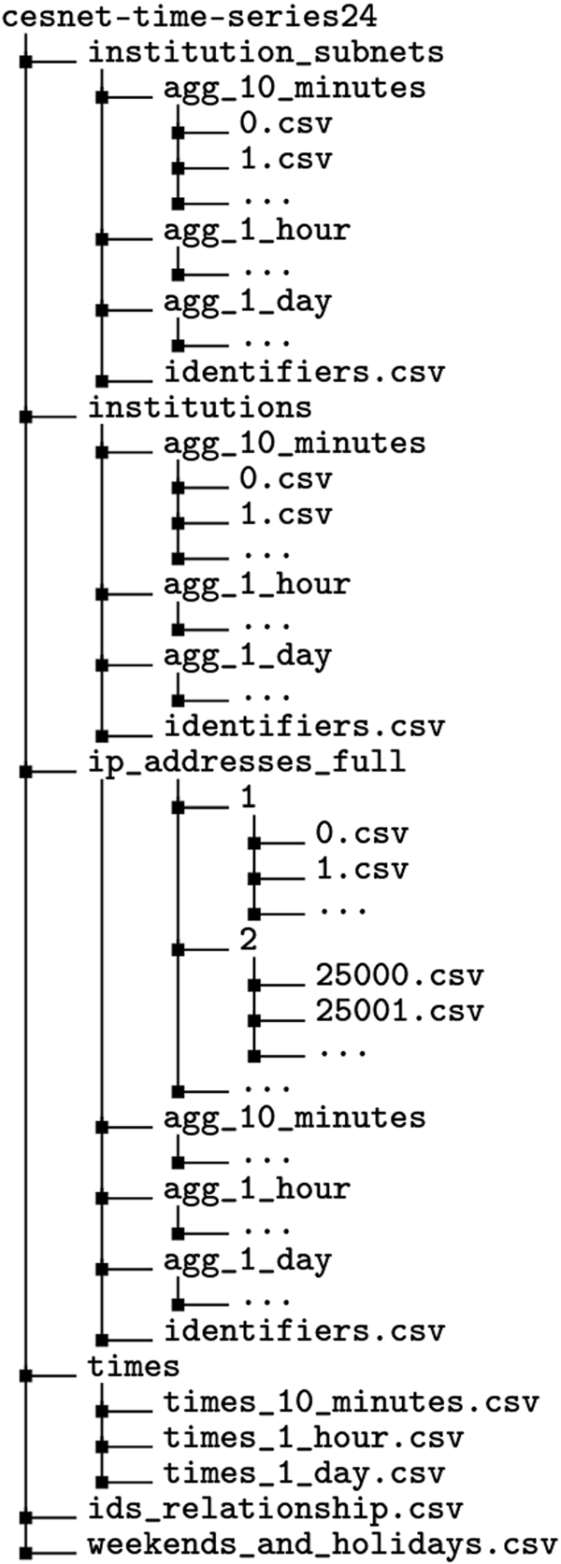
The file structure of the CESNET-TimeSeries24 dataset^14^.

The dataset is available on the Zenodo Platform^14^ in the form of compressed CSV files. The dataset structure is outlined in Fig. 4. Each time series type (either institutional subnets, institutions, or full IP addresses) is also divided by the aggregation time window (10 minutes, 1 hour, and 1 day). The time series data, containing all relevant metrics, is stored in CSV files. Each file is named according to the time series identifier. Due to previous filtration, identifiers are not consecutive, and some numbers are missing. Each time series type has its own identifiers.csv file listing identifiers for all the included entities.

The individual IP address activity time series is additionally divided into multiple subdirectories. Since there are 275,124 IP address time series in the dataset, each located in its own file, some filesystems had difficulty handling such a large number of files in a single directory. Therefore, we organized them into subdirectories, each with 25,000 files. For individual IP addresses, the identifiers.csv file also includes the name of the subdirectory where the corresponding time series data is stored.

We organize the dataset files as tables in CSV file format. There are two different file formats for the time series. The time series aggregated over 10 minutes contain columns described in Table 2. The time series aggregated over one hour and one day contains additional columns described in Table 3.

**Table 2:** Detailed description of time series metrics.

Column name	Description
id_time	Unique identifier for each aggregation interval within the time series, used to segment the dataset into specific time periods for analysis.
n_flows	Total number of flows observed in the aggregation interval, indicating the volume of distinct sessions or connections for the IP address.
n_packets	Total number of packets transmitted during the aggregation interval, reflecting the packet-level traffic volume for the IP address.
n_bytes	Total number of bytes transmitted during the aggregation interval, representing the data volume for the IP address.
n_dest_ip	Number of unique destination IP addresses contacted by the IP address during the aggregation interval, showing the diversity of endpoints reached.
n_dest_asn	Number of unique destination Autonomous System Numbers (ASNs) contacted by the IP address during the aggregation interval, indicating the diversity of networks reached.
n_dest_port	Number of unique destination transport layer ports contacted by the IP address during the aggregation interval, representing the variety of services accessed.
tcp_udp_ratio_packets	Ratio of packets sent using TCP versus UDP by the IP address during the aggregation interval, providing insight into the transport protocol usage pattern. This metric belongs to the interval <0, 1> where 1 is when all packets are sent over TCP, and 0 is when all packets are sent over UDP.
tcp_udp_ratio_bytes	Ratio of bytes sent using TCP versus UDP by the IP address during the aggregation interval, highlighting the data volume distribution between protocols. This metric belongs to the interval <0, 1> with same rule as tcp_udp_ratio_packets
dir_ratio_packets	Ratio of packet directions (inbound versus outbound) for the IP address during the aggregation interval, indicating the balance of traffic flow directions. This metric belongs to the interval <0, 1>, where 1 is when all packets are sent in the outgoing direction from the monitored IP address, and 0 is when all packets are sent in the incoming direction to the monitored IP address.
dir_ratio_bytes	Ratio of byte directions (inbound versus outbound) for the IP address during the aggregation interval, showing the data volume distribution in traffic flows. This metric belongs to the interval <0, 1> with the same rule as dir_ratio_packets.
avg_duration	Average duration of IP flows for the IP address during the aggregation interval, measuring the typical session length.
avg_ttl	Average Time To Live (TTL) of IP flows for the IP address during the aggregation interval, providing insight into the lifespan of packets.

**Table 3:** Time series metrics which replace metrics n_dest_ip, n_dest_asn and n_dest_port in aggregation.

Column name	Description
sum_n_dest_ip	Sum of numbers of unique destination IP addresses.
avg_n_dest_ip	The average number of unique destination IP addresses.
std_n_dest_ip	Standard deviation of numbers of unique destination IP addresses.
sum_n_dest_asn	Sum of numbers of unique destination ASNs.
avg_n_dest_asn	The average number of unique destination ASNs.
std_n_dest_asn	Standard deviation of numbers of unique destination ASNs.
sum_n_dest_port	Sum of numbers of unique destination transport layer ports.
avg_n_dest_port	The average number of unique destination transport layer ports.
std_n_dest_port	Standard deviation of numbers of unique destination transport layer ports.

The dataset contains a times directory with tables to translate sequence numbers between the aggregation time window and the absolute time. Such translation is often useful for plotting forecasting performance. The ids_relationship.csv file described in Table 4 contains a relationship between IP addresses, institutions, and institution subnets. Table 5 describes the weekends_and_holidays.csv, which specifies non-working days in the Czech Republic (weekends and national holidays).

**Table 4:** Content of the ids_relationship.csv file.

Column name	Description
id_ip	ID of the IP address
id_institution	ID of the institution which own the IP address
id_institution_subnet	ID of the institution subnet in which the IP address belongs

**Table 5:** Content of the weekends_and_holidays.csv file.

Column name	Description
Date	The date of the day in format “YYYY-MM-DD”
Type	The type of the day (Weekend or Holiday)

## Technical Validation

This section provides technical validation of the dataset and is divided into three parts: i) Validation of overall dataset properties, ii) Validation of the existence of anomalies, and iii) Validation of usability of the dataset for forecasting approaches. We worked closely in dataset validation with CESNET staff, who had access to the anonymization mapping table in the database framework. They helped us to investigate suspicious phenomena identified in the data as part of their cyber-defense workflow.

### Validation of overall dataset properties

In this section, we aim to validate the overall statistical properties of the dataset across the 40 weeks.

#### Evolution of transmitted data

**Fig. 5:**
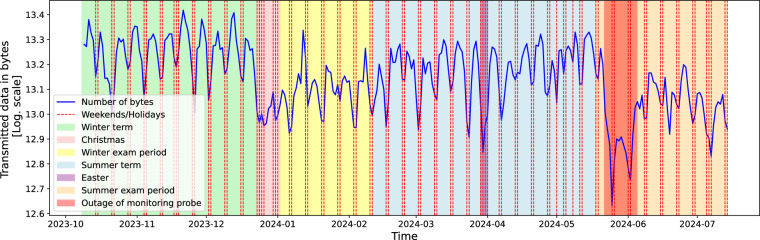
Data transmitted on the CESNET3 network and captured in the dataset.

Data transmitted by IP addresses as captured in the dataset are shown in Fig. 5. Daily transmitted data correlates with weekends and holidays. CESNET3 interconnects universities and dormitories; thus, we can also see a correlation between the terms and the exam periods. This observation is in line with the findings of Beneš *et al*.^22^.

The decrease in traffic after the end of the summer term is larger than the decrease in traffic during Christmas, when we usually monitor the lowest network activity on the CESNET3 network. On further evaluation, we found that one of the monitoring probes was broken from approximately 21. May 2024, 16:30 to approximately 4. June 2024, 20:00. This means that the probe did not send IP flows to the IP flow collector during that time, causing a more noticeable decrease in traffic after the summer term.

#### Gaps in time series

Real-world network traffic data often contains gaps where a device does not transmit any data. In some instances, the entire network’s traffic may exhibit such gaps. These gaps, or spaces, must be addressed before a dataset user applies forecasting methods.

One way to manage these gaps is through aggregation. If the aggregation window is sufficiently large, for example, one day, it can eliminate these gaps from the resulting time series. However, for scenarios involving multiple processes, such as traffic from multiple devices or entire networks, it’s unlikely that a single aggregation window will be effective at every moment. Additionally, using a large aggregation window can obscure patterns in the time series, such as anomalies. As a result, inevitably, some time series will contain gaps.

**Fig. 6:**
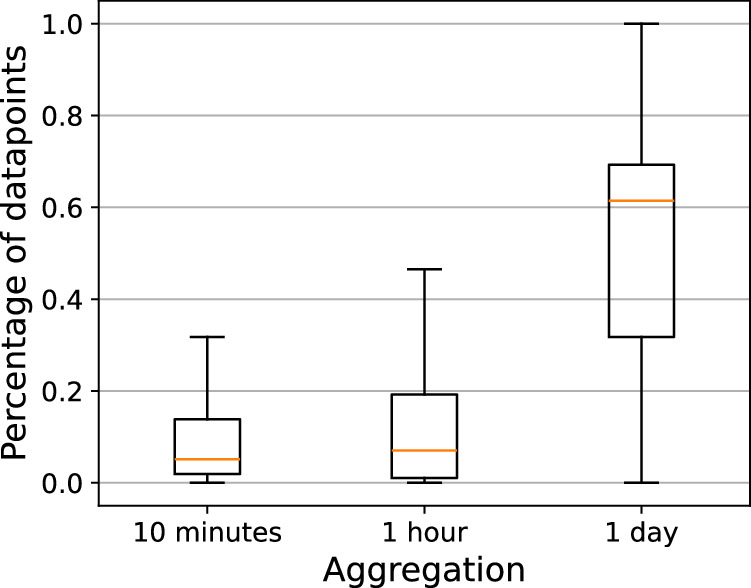
Analysis of datapoints and gaps in dataset using boxplot.

Table 6 shows the percentage of time series in our dataset containing gaps. Figure 6 shows the distribution of datapoints. For the 10-minute aggregation interval, on average datapoints make up less than 20% of the time series, which means that gaps constitute more than 80%. As expected, the number of gaps decreases as the aggregation interval increases. However, even with a day-long aggregation interval, gaps still account for more than 89% of the IP address time series and 6% of the institutional subnet time series.

**Table 6:** Percentage of time series with gaps.

Dataset part	Aggregation		
	10 minutes	1 hour	1 day
IP addresses	99.94%	99.21%	89.81%
Institution subnets	86.31%	31.75%	6.75%
Institutions	77.81%	21.83%	3.16%

**Fig. 7:**
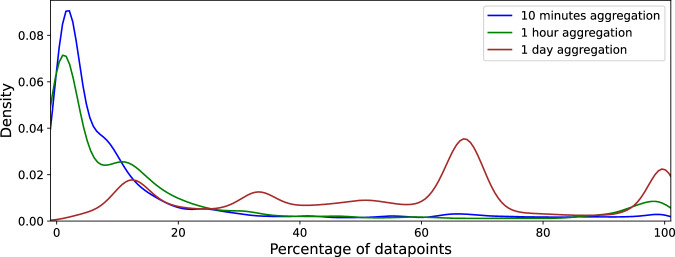
Analysis of datapoints and gaps in dataset using KDE function.

The Kernel Density Estimation (KDE) function in Fig. 7 highlights the same phenomenon as Fig. 6. There is a peak between 60% and 70% of datapoints, which aligns closely with the proportion of working days (67.5%) in the dataset. This pattern suggests that a significant portion of the observed time series activity occurs on working days, which is characteristic of workstations that are primarily used during business hours, as expected for the CESNET3 network.

### Validation of anomalies existence

#### Anomaly types in dataset

**Fig. 8:**
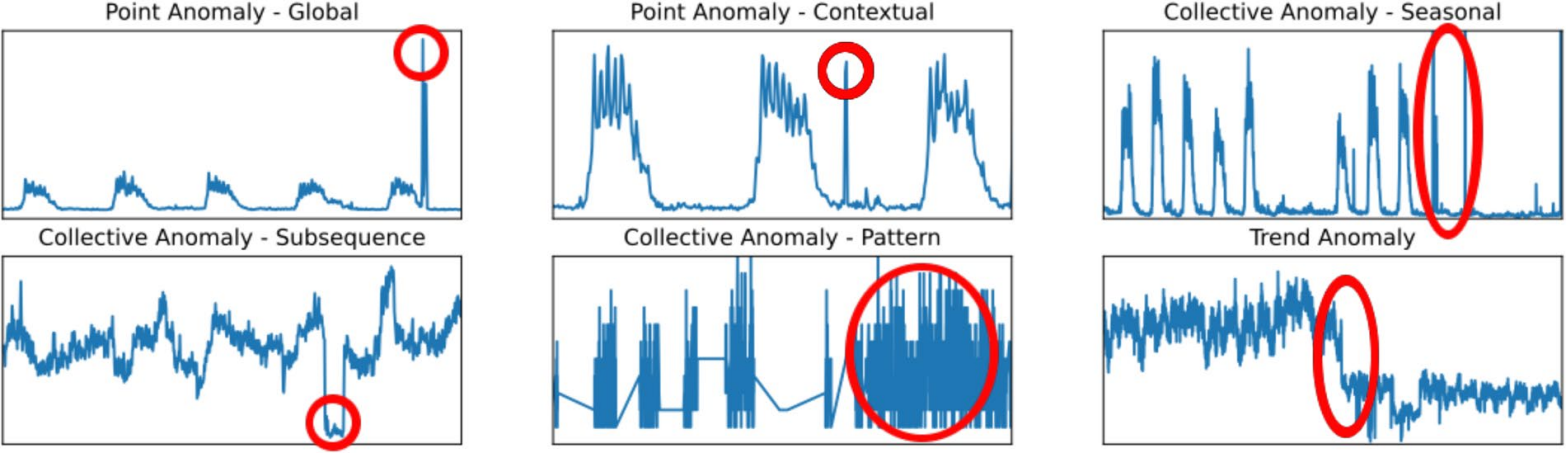
Examples of the anomaly types occurring in the dataset.

There are many types of anomalies described in the literature. Many of these anomalies are present in this dataset. Examples of the anomaly types frequently occurring in the dataset are shown in Fig. 8.

The first type of anomaly is a *Point Anomaly*^15^, a single data point that significantly deviates from the rest of the datapoints in the time series. There are two types of point anomalies:

- Global - A global outlier is a data point that deviates significantly from the overall pattern or distribution of the entire dataset. It is an extreme value when compared to the rest of the data.
- Contextual - A data point that is an outlier within a specific context or condition but not necessarily when viewed in a different context.

The second type of anomaly is a *Collective Anomaly*^15^, a sequence of datapoints that is anomalous when considered together, even if individual points might not be. There are two types of collective anomalies:

- Seasonal - A contiguous subsequence of datapoints that is anomalous compared to the seasonal pattern of the time series.
- Subsequence - A contiguous subsequence of datapoints that is anomalous compared to the rest of the time series.
- Pattern - A sequence of datapoints that together form an unusual pattern that does not conform to known time series patterns.

The third type of anomaly is a *Trend Anomaly*^16^, an unexpected change in the trend of the time series data, such as a sudden shift from a positive to a negative trend. Similar data behaviors in data science are called data or content drifts^23^, and sometimes, this anomaly type is called a *Drift Anomaly*.

#### Security incident analysis

**Fig. 9:**
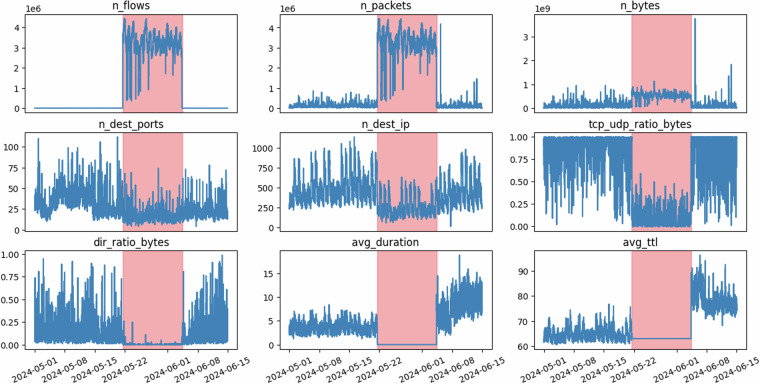
Analysis of detected anomaly on time series IP address ID 1367 identified as DoS by CESNET experts.

When analyzing a detected anomaly from the dataset, it is crucial to explore the metrics that characterize the anomaly. For example, CESNET experts scrutinized metrics in detail for the anomaly detected on time series with IP address ID 1367, as illustrated in Fig. 9, to identify the event as a Denial of Service (DoS) attack.

The analysis reveals several key observations. Both the flow and packet counts (metrics *n_flows* and *n_packets*) increase significantly, yet they rise in parallel, indicating a one-packet flow characteristic. Despite the increase in flows and packets, the transmitted data (metric *n_bytes*) does not rise correspondingly, suggesting that the packets involved are small in size. Furthermore, the number of destination ports (metric *n_dest_ports*) remains steady, dismissing the possibility of a scan. A significant shift in the TCP/UDP ratio (metric *tcp_udp_ratio_bytes*) is observed, with almost all the anomalous traffic being UDP, further supporting the DoS identification.

Additional metrics reinforce this conclusion. The traffic direction (metric *dir_ratio_bytes*) is near zero, indicating nearly all the anomalous traffic is directed toward the monitored IP address. The average duration of the flows (metric *avg_duration*) is close to zero, consistent with one-packet flows typically seen in DoS attacks. The Time To Live (TTL) values (metric *avg_ttl*) remain nearly constant during the anomaly, implying that the traffic likely originates from a single source. Finally, the number of destination IPs (metric *n_dest_ip*) shows a slight decrease, reinforcing the idea of a singular sender and confirming the effectiveness of the DoS attack. These combined metrics clearly point to a DoS event targeting the monitored IP address.

### Validation of dataset usability

We demonstrate the usage of the dataset’s time series for network traffic forecasting to validate the usability of the dataset. We select the time series with IP address ID 103, the number of IP flows, and a one-hour aggregation interval. We used the basic SARIMA (Seasonal Autoregressive Integrated Moving Average) as the forecasting model with the order equal to (1, 1, 1) and seasonal order equal to (1, 1, 1, 168). This means that each of the three components of the model has the same weight in the modeling, and the seasonality was set to one weak (168 hours). The SARIMA was trained on the monthly data (31 datapoints). We use two prediction intervals, 7-days, and 2-days, to demonstrate the difference. The model was continually retrained after each prediction.

**Fig. 10:**
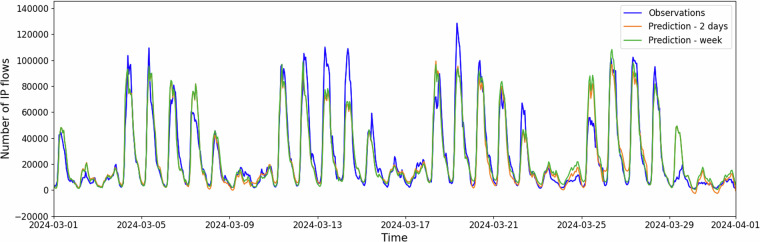
Example of using SARIMA model with order (1, 1, 1) and with seasonal order (1, 1, 1, 168) for forecasting of number of IP flows for time series of IP address’s ID 103.

The result of this forecasting demonstration is shown in Fig. 10. The Figure contains 7-day and 2-day prediction intervals, and the model retraining after 2 days improved results. We compare the results by using the evaluation metrics Root Mean Square Error (RMSE), Symmetric Mean Absolute Percentage Error (SMAPE), and *R*^2^ Score. For all evaluation metrics used, a lower value represents better forecasting performance. The predicted 7-days of data achieves 10,951.26 RMSE, 40.66 SMAPE, and 0.77 *R*^2^ Score. Whereas the predicted 2 days of data achieves 10,293.81 RMSE, 40.86 SMAPE, and 0.79 *R*^2^ Score. In comparison to the predicted 7 day forecast, the predicted 2-day forecast RMSE and *R*^2^ Score were better, but the SMAPE was slightly worse.

## Usage Notes

This section describes recommendations for using the evaluation of network traffic forecasting (and forecasting-based anomaly detection). These recommendations will help the community process our dataset and enable consistent comparisons. Thus, we present a checklist for addressing all our recommendations as shown in Table 7. We have provided an example using the dataset in a Jupyter Notebook on GitHub (https://github.com/koumajos/CESNET-TimeSeries24-Example). The source codes for experiments, including our evaluation example using our recommendations, are also available on this GitHub repository.

**Table 7:** Checklist for Addressing Recommended Evaluation Procedures.

No.	Recommendation
(1)	Specify which dataset(s) are used in the analysis.
(2)	Specify the aggregation interval(s) used.
(3)	Indicate whether the approach is multivariate or univariate.
(4)	Clearly state if not all metrics are used.
(5)	Document all preprocessing steps, including filtering, normalization, and handling gaps in time series.
(6)	Ensure the training phase starts from the beginning of the dataset’s time frame (2023-10-09).
(7)	Specify the duration of the training window.
(8)	Define and describe the validation window if employed.
(9)	Clearly describe the retraining process if the model is retrained during the evaluation phase.
(10)	Specify the forecasting horizon (length of time into the future for predictions).
(11)	Clearly specify the evaluation metrics used in the article.
(12)	Provide an overall comparison across each time series using statistical distributions and aggregate statistics.
(13)	Assess and document the computational requirements and deployability of the model.
(14)	Make source codes of your experiments and model publicly available for the community.

### Dataset selection

The CESNET-TimeSeries24 dataset^14^ is divided into four distinct parts, each of which can be independently used for evaluation: the Full IP addresses, the Sample IP addresses, the Institutions, and the Institution subnets. It is imperative for users to specify the part of the dataset they are using in their analyses *(Recommendation 1)*. Each dataset contains three aggregation intervals: 10 minutes, 1 hour, and 1 day; thus, the aggregation interval(s) used must be specified *(Recommendation 2)*. Where multiple dataset types and/or aggregation levels are employed, results must be reported separately for each dataset and aggregation without combining them to ensure clarity and reproducibility of the findings.

The approach must clearly indicate whether it is multivariate (multiple time series metrics are modeled simultaneously) or univariate (each time series metric is modeled individually) *(Recommendation 3)*. If not all metrics are used, this must be explicitly stated *(Recommendation 4)*. When comparing different methods, ensure that both approaches are either univariate or multivariate so that comparisons are made under the same type of variate conditions. For multivariate approaches, the corresponding method must use the same metrics.

When any preprocessing steps are applied to these dataset parts, it is crucial that these steps are thoroughly described *(Recommendation 5)*. This includes a detailed description of any filtering, normalization, or transformation processes. This also applies to the handling of gaps in time series, which must be addressed and described in detail. Particularly, if the preprocessing involves filtering the time series data, this may lead to results that are not directly comparable with studies using unfiltered versions of the same dataset types. Therefore, such preprocessing steps should be clearly justified, and their impact on the analysis should be discussed.

Given that, the full IP address dataset part contains more than 275,000 time series, evaluating methods for all these time series is challenging. Therefore, we encourage users to create new, smaller datasets featuring interesting time series behaviors from the Full IP address dataset and share them with the community via platforms such as Zenodo. This practice would facilitate further research by providing accessible, focused datasets that highlight specific patterns or anomalies, fostering collaboration and innovation within the research community.

### Training correctness

Users must follow several key guidelines to ensure the integrity and validity of the model training process. The time series training phase must commence from the very beginning of the dataset’s time frame, which starts on 9. October 2023 *(Recommendation 6)*. This allows the model to be trained on the entire range of available data, capturing all relevant trends and patterns and ensuring comparability of performance results. The duration of the training window must be explicitly specified *(Recommendation 7)*. This includes detailing how much historical data is used to train the model before it begins making predictions.

If a validation window is employed during the model development process, the duration and purpose of this validation window must be clearly defined *(Recommendation 8)*. If the model is retrained during the evaluation phase, this retraining process must be clearly described *(Recommendation 9)*. Users should specify the retraining frequency, the data used for retraining, and how the retrained model is validated.

### Forecasting correctness

To ensure consistency and transparency, users must clearly describe the following aspects of their prediction process. Users must specify the length of time into the future predictions are made for *(Recommendation 10)*. This could be a fixed period (e.g., one week ahead) or a rolling window that adjusts over time. If a rolling window is used, users should explain whether the window is shifted by a fixed interval or if it adapts based on certain criteria.

### Evaluation metrics

The forecasting model should be evaluated using evaluation metrics. The chosen metrics must be clearly specified *(Recommendation 11)*. We recommend using the following metrics for evaluation^24^: Root Mean Squared Error (RMSE), Symmetric Mean Absolute Percentage Error (SMAPE), Coefficient of Determination (*R*^2^ score).

### Multiple time series evaluation

All recommended metrics are computed individually for each time series. However, an overall comparison must be made across each time series in the dataset *(Recommendation 12)*. Therefore, we recommend comparisons of overall performance using weighted evaluation metrics (weighted RMSE, SMAPE, or *R*^2^ score) or statistical distributions per metric. Initially, summary statistics such as the mean and standard deviation should provide a general sense of variation across the dataset. For a more detailed evaluation, we suggest plotting distribution plots, such as histograms or Kernel Density Estimation (KDE) plots. KDE plots are highly effective for detailed comparisons across multiple models, offering a nuanced view of the distribution and performance variations.

### Computational requirements and deployability of the model

When evaluating model performance, it is crucial to not only consider its precision but also to assess its computational requirements and the feasibility of the deployment *(Recommendation 13)*. The computational complexity of the model should be analyzed, considering factors such as training time, inference speed, and resource consumption (e.g., CPU/GPU usage, memory footprint). Models that require excessive computational resources may be impractical for real-time applications or deployment in environments with limited resources.

**Table 8:** Software used for creating the dataset.

Name	Version	Link
ipfixprobe	4.11.0	https://github.com/CESNET/ipfixprobe
IPFIXcol2	2.2.1	https://github.com/CESNET/ipfixcol2
NEMEA Framework	0.14.0	https://github.com/CESNET/Nemea-Framework
NEMEA modules	2.20.0	https://github.com/CESNET/Nemea-Modules
NEMEA Supervisor	1.8.2	https://github.com/CESNET/Nemea-Supervisor
TimeScaleDB-14	2.15.0	https://www.timescale.com
Python	3.9.0	https://www.python.org/downloads/release/python-390

## Data Availability

The dataset has been produced using open-source software. The flow exporter ipfixprobe, flow collector IPFIXcol2, the NEMEA processing system, and the NEMEA modules are available on GitHub. We use the TimeScaleDB database. The used open-source software with versions and links to corresponding repositories are summarized in Table 8. We also provide the Create Datapoint module and deployment scripts for the NEMEA Supervisor and for building the database at https://github.com/koumajos/CESNET-TimeSeries24-CD. An example of dataset usage is available at https://github.com/koumajos/CESNET-TimeSeries24-Example.
